# Application of Machine Learning Techniques to High-Dimensional Clinical Data to Forecast Postoperative Complications

**DOI:** 10.1371/journal.pone.0155705

**Published:** 2016-05-27

**Authors:** Paul Thottakkara, Tezcan Ozrazgat-Baslanti, Bradley B. Hupf, Parisa Rashidi, Panos Pardalos, Petar Momcilovic, Azra Bihorac

**Affiliations:** 1 Department of Anesthesiology, College of Medicine, University of Florida, Gainesville, Florida, United States of America; 2 Biomedical Engineering Department, University of Florida, Gainesville, Florida, United States of America; 3 Industrial and Systems Engineering, University of Florida, Gainesville, Florida, United States of America; Massachusetts General Hospital, UNITED STATES

## Abstract

**Objective:**

To compare performance of risk prediction models for forecasting postoperative sepsis and acute kidney injury.

**Design:**

Retrospective single center cohort study of adult surgical patients admitted between 2000 and 2010.

**Patients:**

50,318 adult patients undergoing major surgery.

**Measurements:**

We evaluated the performance of logistic regression, generalized additive models, naïve Bayes and support vector machines for forecasting postoperative sepsis and acute kidney injury. We assessed the impact of feature reduction techniques on predictive performance. Model performance was determined using the area under the receiver operating characteristic curve, accuracy, and positive predicted value. The results were reported based on a 70/30 cross validation procedure where the data were randomly split into 70% used for training the model and the 30% for validation.

**Main Results:**

The areas under the receiver operating characteristic curve for different models ranged between 0.797 and 0.858 for acute kidney injury and between 0.757 and 0.909 for severe sepsis. Logistic regression, generalized additive model, and support vector machines had better performance compared to Naïve Bayes model. Generalized additive models additionally accounted for non-linearity of continuous clinical variables as depicted in their risk patterns plots. Reducing the input feature space with LASSO had minimal effect on prediction performance, while feature extraction using principal component analysis improved performance of the models.

**Conclusions:**

Generalized additive models and support vector machines had good performance as risk prediction model for postoperative sepsis and AKI. Feature extraction using principal component analysis improved the predictive performance of all models.

## Introduction

Postoperative complications are significant sources of morbidity and mortality leading to a multi-fold increase in costs and adverse long-term consequences [1]. Postoperative sepsis and acute kidney injury (AKI) are well-recognized risk factors for short and long term morbidity and mortality after surgery [2–7]. Furthermore, development of AKI during sepsis increases patient morbidity, predicts higher mortality and has a significant effect on multiple organ functions [8]. There is an increasing interest in predicting the probability of postoperative complications in order to improve risk stratification prior to surgery and to allow timely use of preventive therapies during surgery and anesthesia. Assessment of this risk requires timely, accurate and dynamic synthesis of the large amount of clinical information in the preoperative period. Current preoperative risk stratification is limited to a physician’s subjective risk assessment or risk scores that often require elaborate data extraction [9, 10]. While the majority of existing preoperative AKI risk scores are limited to cardiac surgery and have modest accuracy [11, 12], tools for preoperative risk stratification for severe sepsis are missing.

Multivariate regression models are traditionally used for risk prediction in medical research due to their ease of result interpretation and analysis but machine learning classifiers have gained momentum in biomedical research during the past few years with the availability of electronic health records and more complex medical data. Even though the choice of risk prediction model plays a role in generating robust and accurate risk prediction, data cleaning and preprocessing are equally important for model performance [13–15]. There is no consensus about the best choice of mathematical function for predictive models in terms of their performance, and studies have shown relative performance comparisons only on a case-by-case basis [16–25]. Since the dimension of the dataset, including the number and complexity of variables, is an important determinant of the predictive performance, the optimal choice of techniques for reducing data dimensionality is equally important [26].

Using all available preoperative clinical and administrative data in a large retrospective cohort of surgical patients, we studied the effect of data preprocessing, modeling options, and dimensionality reduction on the prediction performance of the models forecasting the risk of postoperative AKI and sepsis prior to surgery.

## Methods

### Source of Data

The study was approved by the UF Institutional Review Board and Privacy Office as exempt study with waiver for informed consent. Using the University of Florida Health Integrated Data Repository as Honest Broker for data de-identification we have created perioperative dataset (DECLARE) that integrated multiple databases within the health system as previously described [4]. Using residency zip code, we have link registry data to the United State Census data [27] to calculate residing neighborhood characteristics and distance from hospital using sp package in R [28]. We included all inpatient operative procedures requiring at least 24 hours hospital stay performed between January 1, 2000 and November 30, 2010.

### Participants

We included all patients with age greater or equal to 18 years admitted to the hospital for longer than 24 hours following any type of inpatient operative procedure. We excluded patients with end-stage renal disease on admission as identified by the previously validated International Classification of Diseases, Ninth Revision, Clinical Modification (ICD-9-CM) diagnostic and procedure codes [29] and those with missing serum creatinine. Final cohort consisted of 50,318 patients.

### Outcomes

Main outcomes were postoperative AKI in the first 7 days after surgery and severe sepsis occurring at any time after surgery. We applied the Kidney Disease Improving Global Outcomes (KDIGO) definition for AKI using serum creatinine changes only without urine output criteria. KDIGO uses either 0.3 mg/dl increase within 48 hours or 50% increase above reference serum creatinine [30]. We followed the criteria of the Agency for Healthcare Research and Quality for the patient safety indicator “Postoperative Sepsis” while organ failure associated with sepsis was identified by adding ICD-9-CM codes for acute organ dysfunction [31, 32].

### Predictor variables

**Fig 1:**
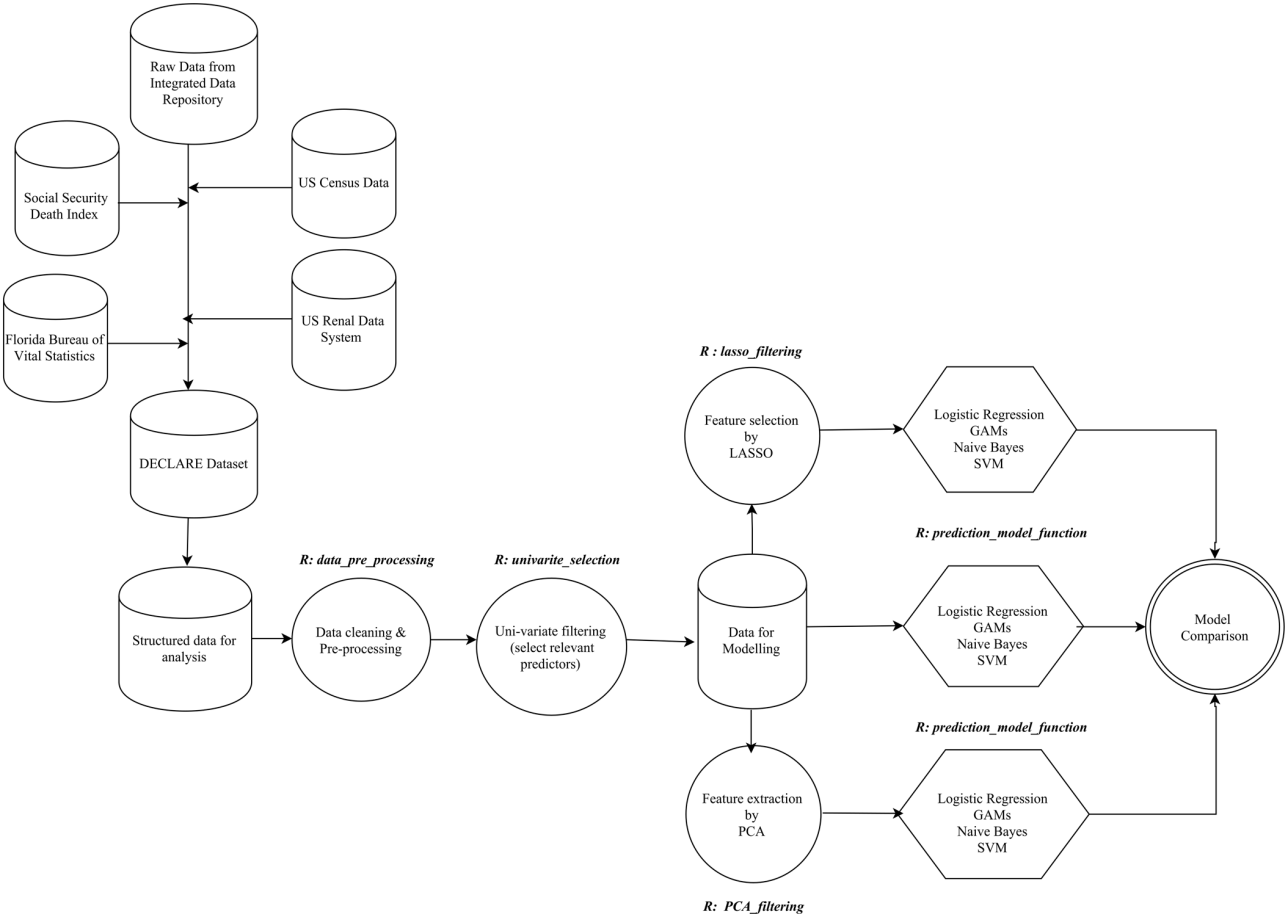
Development flow from raw data to model building. Sequence of steps from aggregation of raw data, data preparation and leading to model building. The R functions used at each stage is represented in bold italics. R functions are available in github repository: https://github.com/PRISMUF/ML_Algorithm_Postoperative.git. GAM, generalized additive model; SVM, support vector machine; LASSO; least absolute shrinkage and selection operator; PCA, principal component analysis.

From the linked DECLARE dataset we used 285 demographic, socio-economic, administrative, clinical, pharmacy and laboratory variables to derive variables for the initial “Preoperative Features Dataset” (Fig 1 and Table 1). Patient comorbidity data was derived using up to 50 ICD-9-CM diagnostic codes recorded in the raw data for each patient. We used the method of Elixhauser et al. to calculate multiple binary comorbidity variables with the exception for chronic kidney disease for which we used updated definitions [29, 33]. Since some comorbidities had low prevalence in the study population (<2%), we included Charlson comorbidity index as a composite measure for medical comorbidities [34] in all multivariable analyses. We extracted medications dispensed on the first admission day using RxNorms data grouped into drug classes according to the US, Department of Veterans Affairs National Drug File-Reference Terminology [35]. For each patient we considered all potential predictors available in the preoperative setting and the final predictor subset for multivariate model was selected by including predictors with statistical significance (P < 0.2) in univariate regression analysis (R function, univariate_selection).

**Table 1:** Characteristics of input variables.

Variable	Type of Variable	Data Source	Number of categories	Type of Preprocessing
**Demographic variables**				
Age (years)	Continuous	Derived		Imputation of outliers^a^; Nonlinear function^b^
Gender	Binary	Raw	2	
Race	Nominal	Raw	5	Optimization of categorical features^c^
**Socioeconomic variables**				
Primary Insurance	Nominal	Raw	4	Optimization of categorical features^c^
Residency area characteristics				
Zip code	Nominal	Raw	10,000	Transformation through link to Census data^d^
County	Nominal	Raw	71	Optimization of categorical features^c^
Rural area	Binary	Derived	2	
Total Population	Continuous	Derived		Obtained using residency zip code with linkage to US Census data^d^; Imputation of outliers^a^
Median Income	Continuous	Derived		Obtained using residency zip code with linkage to US Census data^d^; Imputation of outliers^a^
Total Proportion of African-Americans	Continuous	Derived		Obtained using residency zip code with linkage to US Census data^d^; Imputation of outliers^a^
Total Proportion of Hispanic	Continuous	Derived		Obtained using residency zip code with linkage to US Census data^d^; Imputation of outliers^a^
Population Proportion Below Poverty	Continuous	Derived		Obtained using residency zip code with linkage to US Census data^d^; Imputation of outliers^a^
Distance from Residency to Hospital (km)	Continuous	Derived		Calculated using residency zip code; Imputation of outliers^a^
**Operative characteristics**				
Day of admission	Nominal	Derived	12	Optimization of categorical features^c^
Month of admission	Nominal	Derived	12	Optimization of categorical features^c^
Weekend admission	Binary	Derived	2	
Attending Surgeon	Nominal	Raw	520	Optimization of categorical features^c^
Admission Source	Nominal	Raw	3	Optimization of categorical features^c^
Admission Type	Binary	Raw	2	
Admitting service type	Binary	Derived	2	
Time of surgery from admission (days)	Continuous	Derived		Imputation of outliers^b^
Surgery Type	Nominal	Derived	12	Optimization of categorical features^c^
Primary surgical procedure	Nominal	Derived	1555	Forest tree analysis of ICD-9-CM codes^e^
**Comorbidities**				
Charlson's comorbidity index	Nominal	Derived	18	Optimization of categorical features^c^
Major Diagnosis Category	Nominal	Raw	28	Optimization of categorical features^c^
Myocardial Infarction	Binary	Derived	2	
Congestive Heart Failure	Binary	Derived	2	
Peripheral Vascular Disease	Binary	Derived	2	
Cerebrovascular Disease	Binary	Derived	2	
Chronic Pulmonary Disease	Binary	Derived	2	
Connective Tissue Disease-Rheumatic Disease	Binary	Derived	2	
Diabetes	Binary	Derived	2	
Cancer	Binary	Derived	2	
Liver Disease	Binary	Derived	2	
Chronic kidney disease stage	Binary	Derived	2	
**Medications**				
Indicator of receiving Aminoglycosides on admission day	Binary	Derived	2	
Indicator of receiving Bicarbonate on admission day	Binary	Derived	2	
Indicator of receiving Diuretics on admission day	Binary	Derived	2	
Indicator of receiving Steroid on admission day	Binary	Derived	2	
Indicator of receiving Vancomycin on admission day	Binary	Derived	2	
Indicator of receiving ACE Inhibitors on admission day	Binary	Derived	2	
Indicator of receiving NSAIDS on admission day	Binary	Derived	2	
Indicator of receiving Aspirin on admission day	Binary	Derived	2	
Indicator of receiving Antiemetic on admission day	Binary	Derived	2	
Indicator of receiving Betablokers on admission day	Binary	Derived	2	
Indicator of receiving statin on admission day	Binary	Derived	2	
Indicator of receiving naloxone on admission day	Binary	Derived	2	
Indicator of receiving pressors on admission day^f^	Binary	Derived	2	
Indicator of receiving inotropes on admission day ^f^	Binary	Derived	2	
**Preoperative laboratory results**				
Reference serum creatinine	Continuous	Derived		Imputation of outliers^b^
Reference estimated glomerular filtration rate	Continuous	Calculated from baseline creatinine		Imputation of outliers^b^; Nonlinear function^b^
MDRD creatinine	Continuous	Derived		Imputation of outliers^b^
Ratio of reference creatinine to MDRD Cr	Continuous	Derived		Imputation of outliers^b^
Hematocrit	Continuous	Raw		Imputation of outliers^b^; Nonlinear function^b^
Hemoglobin, g/dl	Continuous	Raw		Imputation of outliers^b^; Nonlinear function^b^
Urine protein, mg/dL	Nominal	Raw	4	Optimization of categorical features^c^
Urinal Hemoglobin, mg/dL	Nominal	Raw	5	Optimization of categorical features^c^
Urinal Glucose, mg/dL	Nominal	Raw	5	Optimization of categorical features^c^
No of complete blood count tests	Nominal	Raw	4	Optimization of categorical features^c^
No of urine tests	Nominal	Derived	4	Optimization of categorical features^c^

^a^ For continuous variables, observations that fell in the top and bottom 1% of the distribution were removed and imputed being considered as outliers.

^b^ Nonlinear risk function was calculated for continuous functions entered to the model.

^c^ For categorical variables with more than two levels, levels were transformed to a numeric value as detailed in Methods section.

^d^ Using residency zip code, we linked to US Census data to calculate residing neighborhood characteristics and distance from hospital.

^e^ Surgical procedure codes were optimized using forest tree analysis of ICD-9-CM codes as detailed in Methods section.

^f^ Variable entered into model as 'Indicator of receiving pressors or inotropes on admission day".

### Sample Size

We included all patients that satisfied our inclusion criteria in the cohort. The reported results were calculated from validation cohort derived using a 70/30 cross validation procedure. We estimated that the validation cohort of 15,000 patients allows a maximum width of 95% confidence interval (CI) for area under the curve (AUC) of 0.02 when prevalence of postoperative complication is 5% and 0.02 when prevalence is 40%.

### Predictive Analytics Process

Fig 1 outlines the experimental design of the predictive analytics process. Following data integration we performed data preparation steps to improve computational efficiency and robustness of prediction models. Data preprocessing included data cleaning with removal of outliers, imputation of missing data, and optimization of categorical and nominal variables (Table 1 and Fig 1) [36, 37]. To address the risk of overfitting, data was randomly split into 70% used for training the model and the 30% for validation for each run [38]. Proportion of AKI and severe sepsis were similar in each partition by the sampling design. The analytical and writing plan followed the TRIPOD recommendations [39].

### Data Cleaning

For all variables we developed set of automatic rules for the removal of outliers that were considered unreasonable observations by medical experts. For continuous variables, observations that fell in the top and bottom 1% of the distribution were considered as outliers. Identified outliers were removed and then treated as missing values. All missing observations were imputed before model building using automated algorithm. For nominal variables with missing entries, a distinct “missing” category was created. For continuous variables, the mean value for a given variable was used for imputation.

### Optimization of Categorical Features

For nominal variables (such as surgeon’s ID) and categorical features with more than two levels (Table 1), the values in each level (*x*_*i*_) were replaced with the ratio:

$$\text{log}\left[\text{P}(X_{i}=x_{i}|E=1)/\text{P}(X_{i}=x_{i}|E=0)\right],$$

where *E* = 1 and *E* = 0 represent a positive and negative outcome respectively as previously described (R function, data_pre_processing) [3]. The probability P(*X*_*i*_ = *x*|*E* = *e*) was estimated by $\#\{j:E^{j}=e,\;x_{i}^{j}=x\}/\#\{j:E^{j}=e\}$ where, *E*^*j*^ represents outcomeat level *j* of categorical variable *x*_*i*_ and $\#\{j:E^{j}=e,\;x_{i}^{j}=x\}$ represents the number of cases with *E*^*j*^ = *e* and $x_{i}^{j}=x$. In case of classification trees such substitution gives the optimal splits, in terms of cross-entropy or Gini index. This transformation of categorical predictors into ordered variable provides better performance and a more robust model by reducing the chance of overfitting. However, when the number of unique values in a categorical variable is very large, the categorical predictor gets grouped into partitions of small size, and hence it will be difficult to obtain statistically significant results. A grouping scheme is used to obtain a reliable estimate of P(*X*_*i*_ = *x*|*E* = *e*), such that risk factor categories with fewer than 100 records determined by sensitivity analysis were grouped together and labeled as "other". This "other" group was further split into several subgroups where each subgroup contained categories with similar proportions of patients from different classes [3]. This was achieved by performing k-means clustering on the set of categories in the "other" group. We set the number of clusters to 5.

### Optimization of surgical procedure codes

The types of surgical procedures were determined using the 4-digit primary procedure ICD-9-CM codes. The exisiting ~ 3000 codes are prefix-based on anatomical location of surgery and often lack detailed descriptions of surgical approach. Although they are important features for risk stratification, their high dimensionality renders them challenging for the use in predictive models. In addition, while for some procedure codes only a few patients were encountered in the cohort, the estimation of probabilities by counting the number of such patients in each class would be unreliable. To overcome this issue we combined procedures with small number of patients into groups of procedures based on their similarity according to the ICD-9-CM classification using forest tree approach as previously described (R function, data_pre_processing) [3]. We created a tree where each node *n* corresponds to a certain group of the procedures and is described by a sequence of digits (*S*_*n*_ length varies from 2 to 4); and each successor of a given node has a code generated by adding to *S*_*n*_ one additional digit from the right. For each leaf node, we assigned a number of patients who had a type of surgical procedure described by this node’s code, and for each non-leaf node (such nodes represent general classes of procedures) we assigned a number of patients whose type of surgical procedure belongs to this class. Procedures were aggregated up to the top level of the ICD-9-CM hierarchy (18 basic procedures classes) such that each procedure/group of procedures contained at least 100 patients. The value of this parameter was selected based on a grid search through the values 50, 100, 150, 250 and 500. We enumerated the obtained set of procedures or groups of procedures and the enumeration index was taken as a discrete feature in our model. The grouping method reduced the number of levels in procedures from 1,536 to 187 and improved the proportion of low frequency procedures.

### Predictive Models

We compared four predictive modeling approaches: Naïve Bayes, generalized additive model (GAM), logistic regression, and support vector machine (SVM) (R function, prediction_model_function). We choose Naive Bayes as the commonly used type of generative models, a category of predictive models that learns the distribution of the input data, and by using this joint probability predicts the outcome using the Bayes rule [40]. More commonly used category of discriminative models learn a direct map from the input data to the response labels and was represented by logistic regression and GAM [40]. Logistic regression is a commonly used method in medical literature and the predicted risk is either monotonically increasing or decreasing. On the other hand, GAM are additive regression models that can relax the monotonicity assumption of logistic models and offer advantage of estimating non-linear risk functions for continuous variables. We used GAM to estimate non-linear functions for age, reference estimated glomerular filtration rate, hematocrit, and hemoglobin. Support vector machine is one of the widely used advanced machine learning techniques [41].

### Generalized Additive Model

We estimated the probability of outcome (*E* = 1, otherwise *E* = 0) by using a generalized additive model:

$$\text{logit P}(E=1|X=x)=\alpha +\sum_{i=1}^{m}f_{i}(x_{i}),$$ (1)

where *m* is the number of risk factors, *X* = (*X*_1_, …, *X*_*m*_) are the risk factors, *x* = (*x*_1_, …, *x*_*m*_) are the values of these factors, *f*_*i*_ is a nonlinear risk function associated with the *i*th risk factor and *α* is a free term. Nonlinear risk functions *f*_*i*_ were estimated for each feature with cubic splines via a local scoring algorithm [42]. The degrees of freedom for each spline were estimated by maximizing restricted likelihood function [43]. Degrees of freedom characterize a curvature of a spline, with value 1 corresponding to a linear function. Risk predictors with estimated degrees of freedom close to 1 were not smoothed in the final model; instead the original values of risk predictors *x*_*i*_ were used. Therefore, the final model has the following form as in Eq 2 were ${\bar{I}}_{}$ is a set of risk predictors with estimated degrees of freedom close to 1 and *w*_*i*_ is the linear weight of the *i*^*th*^ risk predictors.

$$\text{logit P}(\text{E}=1\;|\text{ X}=\text{x})=\;\alpha \;+\sum_{i\in \bar{I}}w_{\text{i}}*x_{i}+\;\sum_{i\in \underline{I}}f_{\text{i}}(x_{i})$$ (2)

### Logistic Regression

Assuming a linear association between the predictor variables and the logit of each outcome (logarithm of the odds of positive outcome), we applied logistic regression algorithm that uses a weighted least squares algorithm to construct a regression line as the best fit through the data points by minimizing the weighted sum of squared distances to the fitted regression line [21]. Logistic regression model for independent predictors from set *I* is defined as Eq 1, where P(E = 1|X) is the probability of predicting a positive outcome given X and *β*_i_ are the coefficients estimated from data.

$$\text{P}(\text{E}=1\;|\text{ X})=\;\frac{\text{1}}{1\;+{\text{ e}}^{-(\beta_{\text{0}}+\sum_{i\in I}\beta_{i}*x_{i})}}$$ (3)

### Naïve Bayes Model

Naïve Bayes is a probabilistic classifier, based on applying Bayes’ theorem, and assumes that given the class of the outcome vector, the covariates are independent. The probability of event P(E = 1|X = x) is estimated from $\text{P}(\text{E}=1)*\prod_{\text{i}=1}^{\text{m}}\text{P}(\text{X}=\text{x}|\text{E}=1)$. Even though this assumption is not generally true, it simplifies the model complexity and is often seen to outperform other sophisticated alternatives [44].

### Support Vector Machine

SVM is a discriminative model that performs classification by finding a separating decision boundary called “hyperplane” in the input feature space [45–47]. If no linear separation is possible, the SVM algorithm can map the input feature space to a higher dimension using kernel functions and then can construct an optimal separating hyperplane. Consider a binary classification problem with predictors ∈{1,−1} and a hyperplane *wx*−b = 0. A simple SVM model can be represented as minimizing ∥*w*∥ subject to *w* • *x*_*i*_*−b* ≥ 1 for class 1 and *w* • *x*_*i*_*−b* ≤ 1 for class -1. SVM has excellent generalization performance, however compared to the other basic regression techniques the computational cost of training the SVM model is higher and it can be as high as O(n^3^) especially for kernel SVMs.

### Performance Enhancement through Data Reduction Techniques

To overcome the high dimensionality of our dataset we tested two data reduction techniques focused on reducing the size of data. For the first approach, we used the Least Absolute Shrinkage and Selection Operator (LASSO) technique (R function, lasso_filtering [48]) as a feature selection technique to select the best subset of features from the initial dataset. For the second approach, we performed feature extraction with Principal Component Analysis (PCA) technique (R function PCA_filtering), which creates a set of meta-features that are linear combination of the original feature set with first component capturing the largest variance, the second principal component exhibiting the second largest variance, and so on. To avoid overfitting, only the top five principal components were considered for model building and all the principal components were uncorrelated. The compressed data can help in speeding up the algorithms (used for prediction models), and in removing redundancy in data, and thereby improving the model performance.

### Internal Validation

The results were reported based on a 70/30 cross validation procedure where the data were randomly split into 70% used for training the model and the 30% for validation. The process was repeated 50 times to report performance measures and relevant confidence intervals.

### Model Performance

We assessed each model’s discrimination using the area under the AUC and model accuracy by determining the fraction of correct classification and positive predicted value for each model. We used bootstrap sampling to obtain 95% confidence intervals for these statistics, and comparisons were made using nonparametric methods. Model calibration was tested using Hosmer-Lemeshow statistic.

## Results

### Participants

Among 50,318 adult patients who underwent major inpatient surgery, 36% (n = 18246) developed AKI in the first seven postoperative days. The severe sepsis occurred among 5% (n = 2589) of the cohort (Table 2). The distribution of outcomes and preoperative clinical characteristics did not differ between training and validation cohorts.

**Table 2:** Summary of overall cohort. Abbreviations. GFR, Glomerular filtration rate, CBC, complete blood count.

	Overall (N = 50318)
**Demographic features**	
Age, median (25th-75^th^)	56 (43, 68)
Female Gender, n (%)	24670 (49.0)
Race, n (%)	
White	40515 (82.2)
African-American	6183 (12.5)
Hispanic	1534 (3.1)
Other	1064 (2.2)
Primary Insurance Group, n (%)	
Medicare	19469 (38.7)
Medicaid	6518 (13.0)
Private	20592 (40.9)
Uninsured	3736 (7.4)
**Socio-economic features**	
Neighborhood characteristics	
Rural area, n (%)	16098 (32.1)
Total Population, median (25th-75th)	17085 (10002, 27782)
Median Income, median (25th-75th)	33293 (28451, 40309)
Total Proportion of African-Americans, median (25th-75th)	0.10 (0.04, 0.20)
Total Proportion of Hispanic, median (25th-75th)	0.04 (0.02, 0.06)
Population Proportion Below Poverty, median (25th-75th)	0.13 (0.09, 0.19)
Distance from Residency to Hospital (km), median (25th-75th)	53(26, 118)
County (top 3 categories), n (%)	
Alachua	8667 (17.2)
Marion	4807 (9.6)
Lake	2155 (4.3)
**Comorbidity features**	
Charlson's comorbidity index (CCI), median (25th-75th)	1 (0, 2)
Cancer, n (%)	10121 (20.1)
Diabetes, n (%)	8332 (16.6)
Chronic Pulmonary Disease, n (%)	8179 (16.3)
Peripheral Vascular Disease, n (%)	5953 (11.8)
Cerebrovascular Disease, n (%)	4175 (8.3)
Congestive Heart Failure, n (%)	3946 (7.8)
Myocardial Infarction, n (%)	3290 (6.5)
Liver Disease, n (%)	2482 (4.9)
Number of diagnoses, median (25th-75th)	8 (5, 13)
Major Diagnosis Category (top 3 categories), n (%)	
Musculoskeletal System and Connective Tissue	9924 (19.7)
Circulatory System	7507 (14.9)
Nervous System	6675 (13.3)
**Operative features**	
***Admission***	
Weekend admission, n (%)	6895 (13.7)
Admission day (top 3 categories), n (%)	
Tuesday	10174 (19.8)
Wednesday	9065 (17.6)
Monday	8843 (17.2)
Admission month (top 3 categories), n (%)	
March	4549 (9.0)
October	4471 (8.9)
January	4449 (8)
Number of operating surgeons, n	520
Number of procedures per operating surgeon, n (%)	
First rank	1905 (3.8)
Second rank	1602 (3.2)
Third rank	1532 (3.0)
Admission Source, n(%)	
Emergency room	13066 (26.4)
Outpatient setting	29826 (60.1)
Transfer	6699 (13.5)
Emergent surgery status, n (%)	22820 (45.4)
Admission to Surgical service, n (%)	44652 (88.7)
Time of surgery from admission (days), n (%)	
0	28613 (56.9)
1–2	11397 (22.6)
> = 3	10308 (20.5)
Surgery Type, n (%)	
Neurologic Surgery	8385 (16.7)
Orthopedic Surgery	7472 (14.9)
Cardiothoracic Surgery	6755 (13.5)
Trauma/Burn Surgery	5650 (11.2)
General Gastrointestinal Surgery	4120 (8.2)
Transplant Surgery	2765 (5.5)
Urological Surgery	2640 (5.3)
vVascular Surgery	2601 (5.2)
Gynecologic Surgery	2437 (4.8)
General Oncology Surgery	2188 (4.4)
General Colorectal Surgery	1833 (3.6)
Other Surgeries^a^	3472 (6.9)
Surgery procedure type	
Primary Procedure codes, n	1555
Primary Procedure (top 3 categories), n (%)	
01.59 Other excision or destruction of lesion or tissue of brain	1330 (2.6)
81.54 Total knee replacement	1223 (2.4)
39.51 Clipping of aneurysm	1099 (2.2)
**Preoperative and admission day laboratory results**	
Reference creatinine (mg/dl), median (25th-75th)	0.8 (0.7, 1.03)
Estimated reference GFR (mL/min/1.73 m2), median (25th-75th)	92.3 (71.1, 107.9)
Hemoglobin, g/dL median (25th-75th)	11.7 (10.2, 13.2)
Hematocrit, median (25th-75th)	34.3 (30.1, 38.6)
Dipstik urine protein, mg/dL n (%)	
Missing	41948 (83.4)
Negative	5502 (10.9)
30	1756 (3.5)
100	753 (1.5)
> = 300	359 (0.7)
Number of CBC tests, n (%)	
0	14620 (29.1)
1	27554 (54.8)
2	5912 (11.8)
3 or more	2232 (4.4)
**Admission day medications**	
Admission medication types, n	40
Admission Day Medications (top 3 categories), n (%)	
Antiemetic drugs	28783 (57.2)
Beta blockers	11750 (23.4)
Diuretics	5886 (11.7)
Statin	5790 (11.5)
Angiotensin-Converting-Enzyme Inhibitors	5066 (10.1)
Aspirin	3428 (6.8)
Pressors/Inotropes	2864 (5.7)
Bicarbonate	2070 (4.1)
Naloxone	575 (1.1)
**Outcomes**	
KDIGO-AKI	18246 (36%)
Severe sepsis	2589 (5%)

^a^ Other surgeries include ear-nose-throat, ophthalmology, and plastic surgeries.

### Model specification

The data preprocessing significantly improved computational efficiency as measured by the time required for model building for all types of models (Table 3). Both logistic regression and GAM demonstrated the largest improvement as the computational time was reduced by one hundredth in the pre-processing step. As expected the independence assumption of the Naïve Bayes model undermined the effect of our grouping scheme for data pre-processing thus gain in computational efficiency was not as large. The inherent computational intensity of the SVM model prevented this comparison as SVM algorithm took on average 2 to 3 hours for one simulation.

**Table 3:** Comparison of time required for model building in seconds.

	Acute Kidney Injury		Severe Sepsis	
	Time required for model building (in seconds)			
Model	Before data preprocessing or optimization	After data preprocessing or optimization	Before data preprocessing or optimization	After data preprocessing or optimization
Logistic Regression Model	4640 s	7 s	5530 s	8 s
Generalized Additive Models	6520 s	48 s	6980 s	73 s
Naïve Bayes Model	26 s	22 s	19 s	24 s

### Model performance

**Fig 2:**
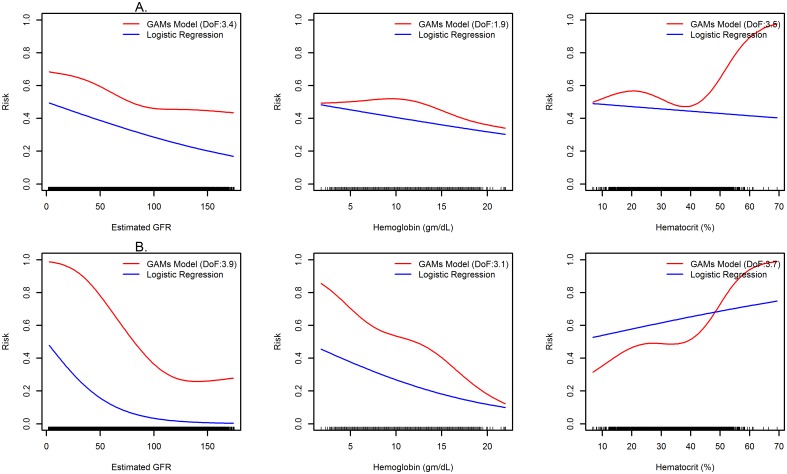
Predicted risk functions for the association between (A) acute kidney injury and (B) severe sepsis and continuous variables. Risk functions were generated from multivariate generalized additive models and logistic regression models. GAM, generalized additive model; DoF, degree of freedom; GFR, glomerular filtration rate.

Table 4 compares the predictive performance of different modeling approaches. Both GAM and logistic regression had improved performance and model fit compared to Naïve Bayes model with all AUCs above 0.80 and between 0.022 and 0.03 higher for predicting AKI and severe sepsis, respectively. Although discriminative performance of GAM was not significantly higher than logistic regression, they were able to account for the non-linearity of continuous clinical variables. Risk patterns for the plasma hematocrit, hemoglobin and estimated glomerular filtration rate showed clearly how non-linear models can effectively depict the risk variation compared to linear models (Fig 2).

**Table 4:** Comparison of model performances. Abbreviations. AUC, area under the receiver operating characteristics curve; CI, confidence interval; GAM, generalized additive model; SVM, support vector machine; LASSO; least absolute shrinkage and selection operator; PPV, positive predicted value. Bootstrap sampling was used to obtain 95% confidence intervals and comparisons were made using nonparametric methods.

Model	Acute Kidney Injury			Severe Sepsis		
	Accuracy (95% CI)	AUC (95% CI)	PPV (95% CI)	Accuracy (95% CI)	AUC (95% CI)	PPV (95% CI)
**Logistic Regression Model**	0.752 (0.746,0.758)	0.824 (0.818,0.828)^b^	0.725 (0.714,0.737)	0.773 (0.762,0.781)	0.851 (0.840,0.8560)	0.811 (0.785,0.833)
**GAMs**	0.756 (0.751,0.761)	0.827 (0.821,0.832)^a^	0.719 (0.706,0.729)	0.775 (0.766,0.783)	0.852 (0.840,0.863)	0.806 (0.779,0.832)
**Naïve Bayes Model**	0.744 (0.738,0.749)	0.797 (0.791,0.803)^a^^,^^b^	0.545 (0.534,0.558)	0.805 (0.798,0.811)	0.83 (0.819,0.841)^a^^,^^b^	0.689 (0.659,0.716)
**SVM**	0.767 (0.757,0.774)	0.819 (0.811,0.828)^a^^,^^b^	0.662 (0.648,0.676)	0.71 (0.689,0.731)	0.762 (0.733,0.782)^a^^,^^b^	0.677 (0.619,0.722)
**After feature selection with LASSO**						
**Logistic Regression Model**	0.753 (0.747,0.757)	0.824 (0.818,0.830)^b^	0.726 (0.714,0.738)	0.772 (0.760,0.780)	0.85 (0.838,0.863)^b^	0.812 (0.781,0.838)
**GAMs**	0.757 (0.752,0.762)	0.828 (0.822,0.833)^a^	0.72 (0.706,0.732)	0.774 (0.766,0.780)	0.851 (0.842,0.862)	0.806 (0.783,0.831)
**Naïve Bayes Model**	0.744 (0.737,0.750)	0.797 (0.789,0.804)^a^^,^^b^	0.545 (0.533,0.556)	0.806 (0.800,0.813)	0.831 (0.817,0.841)^a^^,^^b^	0.69 (0.659,0.711)
**SVM**	0.767 (0.759,0.774)	0.82 (0.812,0.829)^a^^,^^b^	0.665 (0.646,0.685)	0.697 (0.684,0.713)	0.757 (0.736,0.779)^a^^,^^b^	0.689 (0.652,0.732)
**After feature extraction with 5 principal components**						
**Logistic Regression Model**	0.774 (0.769,0.781)	0.853 (0.849,0.859)^a^^,^^b^	0.758 (0.746,0.767)	0.818 (0.809,0.824)	0.904 (0.895,0.913)^a^^,^^b^	0.854 (0.841,0.880)
**GAMs**	0.773 (0.768,0.777)	0.858 (0.853,0.862)^a^^,^^b^	0.784 (0.771,0.793)	0.826 (0.819,0.833)	0.909 (0.902,0.917)^a^^,^^b^	0.86 (0.843,0.878)
**Naïve Bayes Model**	0.741 (0.735,0.747)	0.819 (0.814,0.826)^a^^,^^b^	0.666 (0.651,0.677)	0.805 (0.797,0.815)	0.882 (0.874,0.890)^a^^,^^b^	0.839 (0.822,0.866)
**SVM**	0.777 (0.767,0.782)	0.857 (0.850,0.862)^a^^,^^b^	0.735 (0.725,0.750)	0.85 (0.737,0.897)	0.877 (0.828,0.904)^a^^,^^b^	0.751 (0.667,0.850)

^a^ p<0.05 for AUC comparison with respect to logistic regression model without any data reduction.

^b^ p<0.05 for AUC comparison with respect to GAMs model without any data reduction.

Table 4 details the performance enhancement achieved using data reduction techniques. Using LASSO to reduce the input feature space had minimal effect on prediction performance, while data reduction using principal component analysis improved the models predictive power. Models built using the first 5 principal components provided a 3–6% enhancement in AUC, 2–3% improvement in accuracy and 7–10% improvement in positive predictive values for GAM and similar trend was observed for logistic regression model, SVM and Naïve Bayes model. After application of principal component analysis the predictive performance of GAM and SVM models was comparable.

## Discussion

Our study demonstrates that application of data preprocessing, choice of predictive modeling approach and dimensionality reduction techniques affect the risk prediction performance for two major postoperative complications using routinely available data in electronic health records. Data processing techniques had a positive impact on computational efficiency, and data reduction techniques improved the model predictive capability. Model comparison revealed GAM and SVM as the best options for building risk prediction models among four approaches we used since they provided high discrimination while accounting for the non-linearity of continuous clinical variables.

We developed models that could be applied at point of access to preoperative care, do not rely on self-reported data and specialized testing and were derived from whole population data that are routinely collected in preoperative period. Our data set included readily available variables in electronic health records, many of which are complex variables with multiple values such as residency ZIP codes, procedure and diagnostic codes and surgeon’s identities. Inclusion of these non-traditional variables in the model allowed us to capture certain aspects of health discriminators such as socio-economic status or effect of individual surgeon’s performance. The importance of ZIP code as a surrogate of neighborhood socio-economic characteristics has been recently brought to light by studies demonstrating its powerful association with multiple disease and health behaviors, including obesity, smoking, depression, heart disease and cancer [49–54]. The effect of the performance of surgeon or anesthesiologist is increasingly recognized as an important predictive factor of postoperative outcomes yet it remains controversial and is not routinely incorporated in publicly available risk calculators [55–57].

The data cleaning step is focused on improving the quality of data to make them “fit for use” by users, through reducing errors in the data by removing noise and outliers and improving their documentation and presentation [58]. Since data error rate of 1–5% can be expected, detailed and methodological processing of raw data is a crucial step before performing any analysis to reduce the influence of Type I and Type II errors. The improvement in computational efficiency that was achieved in our study with data cleaning and preprocessing, emphasizes the need for appropriate techniques to process data on a case-by-case basis. Designing data preparatory steps should be based on a good understanding of the nature of data and the clinical needs. In this study we developed a data processing methodology based on the distribution of each variable and its clinical background and relevance. Further, the robustness and smaller error margin of performance measures for all the models in our study can be partly credited to the efficient data preparatory steps.

Even though we did not observe statistically significant difference among model performances, GAM was the preferred model in this study due to its accuracy, relative efficiency compared to SVM and ability to account for non-linearity of variables. GAM is a data driven prediction model that has the flexibility to capture non-monotonicity in the predicted risk and this could be a contributing factor for GAM to gain better results compared to the other prediction models. GAM as a non-linear additive algorithm has generated better fit in comparison to the other prediction models. Since parameter tuning is critical for SVM further fine tuning of SVM parameters may potentially improve the current results. Comparison of model performance with respect to various prediction models shows a performance saturation trend. Our results confirm previous findings that data reduction techniques may improve model performance with principal component analysis providing the best results for our models [59].

With the availability of electronic health records the increasing use of prediction models utilizing these data can be expected. Such models can be a useful guide for healthcare experts to identify patients who may benefit the most from interventions that can mitigate such risks. For patients, these models may provide tools to facilitate informed decision about surgical procedures and risk of complications. To be useful for these purposes, a prediction model must provide validated, robust and accurate estimates. All the prediction models compared in this study provided satisfactory performance in accuracy of the individual risk prediction that will require further external and prospective validation.

## Conclusions

Using predictive analytics and machine learning approaches we have built robust predictive models forecasting risk of two major postoperative complications. Generalized additive models and support vector machines showed superior performance compared to the other models selected for this study. These models could be applied at point of access to preoperative care, do not rely on self-reported data and specialized testing and were derived from whole population data that are routinely collected in preoperative period. We provide set of data preparatory steps to ensure performance and computational efficiency. Choice of predictive function and dimensionality reduction techniques are important to optimize the performance of risk models and further comparison of different machine learning approaches is warranted. In our analysis, feature extraction using principal component analysis improved the predictive performance of all models. External prospective validation of these models is necessary prior to implementation in real-time clinical workflow for automated and simplified risk stratification in preoperative period.
